# Molecular Diagnosis and Cancer Prognosis—A Concise Review

**DOI:** 10.3390/diagnostics13040766

**Published:** 2023-02-17

**Authors:** Thatchanamoorthy Thenrajan, Subbiah Alwarappan, Jeyaraj Wilson

**Affiliations:** 1 Polymer Electronics Lab., Department of Bioelectronics and Biosensors, Alagappa University, Karaikudi 630003, Tamil Nadu, India; 2 CSIR-Central Electrochemical Research Institute, Karaikudi 630003, Tamilnadu, India

**Keywords:** cancer biomarkers, noninvasive, nucleic acid, protein, diagnosis

## Abstract

Cancer is a complicated disease. Globally, it is one of the major causes for morbidity and mortality. A critical challenge associated with it is the difficulty to accurately diagnose it at an early stage. The malignancy due to multistage and heterogeneity that result from genetic and epigenetic modifications poses critical challenge to diagnose and monitor the progress at an early stage. Current diagnostic techniques normally suggest invasive biopsy procedure that can cause further infections and bleeding. Therefore, noninvasive diagnostic methods with high accuracy, safety and earliest detection are the needs of the hour. Herein, we provide a detailed review on the advanced methodologies and protocols developed for the detection of cancer biomarkers based on proteins, nucleic acids and extracellular vesicles. Furthermore, existing challenges and the improvements essential for the rapid, sensitive and noninvasive detection have also been discussed.

## 1. Introduction

Generally, the detection of biomarkers in oncology for diagnosis, screening prognosis, observation of disease is effective for controlling the infections in health care sector. At various stages of cancer, the validation of drugs, evaluation and drug response is carefully monitored, for which antigens associated with tumor are used as standard biomarkers. The biological fluids from the patients such as blood, urine and biopsy samples will be tested to confirm the presence of cancer. Biomarkers may exist on the surface or within the cells and provide extensive information regarding the diseased state. Though there are different types of cancer treatments including conventional surgery, chemotherapy, immunotherapy, and radiotherapy are available due to the poor efficacy of drugs at the targeted site and their associated side effects, these therapeutic treatments are not very attractive.

Despite there being plenty of known techniques available for solid tumors detection, highly advanced strategies are still essential to track disease onset, analyze response in patients to improve prognosis, enhance quality life and to improve the recovery rate in patients. For example, microfluidic technique is a powerful strategy that can perform difficult operations very rapidly with the need of only a small number of reagents. However, the diagnosis of cancer patients with very high precision is limited by isolation, reliable capture and cell enumeration. Liquid biopsy is also a widely preferred technique which is alternate to conventional tissue-based biopsy. The advantages of this technique include rapid analysis time, minimum sample requirement and non-existence of the interpretation issues in comparison with tissue samples. The development of biosensors for the early cancer diagnosis is of great significance as these techniques offer highly sensitive, selective and rapid point-of-care detection.

### Rapid Point-of-Care Detection

Recently, molecular cancer diagnosis using biomarkers measure the related information of genes and proteins of cancer patients for diagnosis and prognosis. The rapid diagnosis of these infectious pathogens is crucial to minimize the huge cancer outbreak. Without any symptoms, massive screening and early diagnosis can control the morbidity to increase the human life expectancy. Of the various known biomarkers, metabolites, proteins and nucleic acid are so attractive because they provide rich information about the diseased state and offer a great opportunity for the early diagnosis and progress of treatments. Metabolites are small molecules that represent the biological processes happening in tissues, cells and biofluids which provides more insights into the mechanisms of disease process and its progression. Furthermore, metabolites provide information such as circulation that occurs in the central nervous system. Interestingly, the circulating metabolites can be released into the extracellular environment as free molecules, or inside extracellular vesicles thereby making it possible for disease detection. Thus, metabolites can be followed as potential biomarkers for cancer detection.

Countries with poor medical care facilities suffer huge mortality rates due to cancer. Therefore, there is a pressing need to develop a low-cost diagnostic tool to detect cancer with high sensitivity and selectivity. Early-stage cancer diagnosis is essential for the treatment and disease management. Molecular level changes provide valuable information about the individuals with risk and development of the disease by the cellular processes, i.e., the changes in expression of DNA, RNA and protein can differentiate healthy and cancer patients. The genomic modifications of DNA and RNA offer new perspective about the functional mechanisms of nucleic acids in cancer, aptamers and DNAzymes. As a result, these are also novel markers for cancer diagnosis. In molecular diagnosis, extracellular vesicles also received significant attention for biomarker discovery. Extracellular vesicles are well-known for biological effects such as signaling and transfer of cargo, influencing the immune response, cell-to-cell communication. Moreover, it is interesting to note that the extracellular vesicles are highly stable at different pH, possibility of long-term storage under adverse physical conditions and good encapsulation behavior in biological fluids such as DNA, RNA binding complexes. All these attributes make them appealing for biomarker development. Furthermore, information about extra cellular vesicles derived from tumor cells at different stages of cancer will be useful to develop innovative diagnostic tool by using the body fluids.

Biosensors are considered as point-of-care (POC) devices due to their ability to analyze clinical samples at home or in a doctor’s office. Biosensors offer cutting-edge platforms for biomarker analysis with the benefits such as simple to use, affordable, quick, and robust, in addition to its ability to test many analytes simultaneously [1]. It is well known that most cancers are associated with the secretion and release of more than one biomarker. Therefore, development of biosensors that can detect several analytes may be helpful for the diagnosis and monitoring of cancer. The simultaneous detection of several markers not only aids in diagnosis but also saves time and money [2]. Very often, electrochemical biosensors are reported for the detection of cancer biomarkers. In the development and design of various electrochemical biosensors, nanostructured materials and nanocomposites play a crucial role, and their utilization has increased recently [3]. For the detection of antibodies, optical biosensors are preferred and widely reported. Due to its direct signal translation mechanism, optical imaging is also more suited for in-vivo sensing applications [4]. Furthermore, surface plasma resonance (SPR) has grown to be a popular sensing technology with many applications due to its ability for a highly sensitive real-time monitoring of analyte-analyte interactions. Typically, SPR devices monitor the changes in resonance angle or wavelength to acquire information about biomolecular interaction [5]. Microfluidic systems are anticipated to emerge as a critical technology for cancer diagnosis and prognosis, although not being used in clinical settings till date. To avoid the use of tumor tissue biopsies, microfluidic devices have been designed for the investigation of a variety of biomarkers including circulating tumor cells, cell-free DNA, exosomes, and proteins, largely in liquid biopsies such as serum, plasma, and whole blood [6]. All these techniques are evidence for the indispensable role of biosensors in cancer diagnosis.

## 2. Biomarkers

According to National Cancer Institute (NCI), biomarkers are defined as “a biological molecule found in blood, other body fluids, or tissues that provides information about a normal or abnormal process, or of a condition or disease”, such as cancer [7]. Biomarkers often distinguish a patient from a healthy individual. Numerous variables, including germline or somatic mutations, transcriptional changes, and posttranslational modifications might cause the changes. Biomarkers exist as proteins (such as an enzyme or receptor), nucleic acids (microRNA or other non-coding RNA), antibodies, and peptides. A biomarker can also be a group of changes, including gene expression, proteomic and metabolomics patterns. Biomarkers are used to predict prognosis or the chance of disease recurrence in cancer patients without the need for treatment. The clinical pathological characteristics of a tumor have traditionally been utilized to determine prognosis. More recently, prognosis for specific malignancies has been determined using modern methods. Most importantly, the impact level in the highly cited article was, in the vast majority of situations, much larger than that in either larger research of the same marker (86% of the time) or a meta-analysis (83%). Upon evaluating the use of a biomarker, it is crucial that researchers objectively assess the literature rather than depending only on a reference to a regularly published study in a review [8].

### Different Types of Cancer Biomarkers

Some biomarkers are common for certain type of cancers. For example, biomarkers related to cancers in the kidney, pancreas, thyroid, and bladder are prostate-specific antigen (PSA), messenger RNA (mRNA), cancer antigen 125 (CA-125), and CA19-9. For blood, immune system and liver-based cancers, B-cell leukemias and lymphomas (BCL), Tyrosine-protein kinase (ABL), cluster of differentiate (CD) 20 antigen, CD 30, and Thiopurine methyltransferase (TPMT) are used. Biomarkers for colorectal, lung, and prostate cancers include the estrogen receptor (ER), progesterone receptor (PR), human epidermal growth factor receptor 2 (HER2), anaplastic lymphoma kinase (ALK), and Kirsten rat sarcoma (KRAS).The bone and muscle cancers have breast cancer genes 1 and 2 (BRCA1 and BRCA2), echinoderm microtubule-associated protein-like 4 (EML4), and carcino-embryonic antigen (CEA) as their biomarkers. On the other hand, some biomarkers are very much specific to one type of cancer only. For example, prostate-specific antigen (PSA) is affiliated with prostate cancer, human epididymis protein 4 (HE4) is related with ovarian cancer, alpha-fetoprotein (AFP) is associated with liver cancer, carcinogenic embryonic antigen (CEA) is linked with colon cancer, thyroglobulin (Tg) is associated with thyroid cancer, while HER2/NEU and CA15.3/CA27.29 is associated with breast cancer [9,10].

## 3. Protein Biomarkers

Protein biomarkers for cancer detection typically emerge from the cancer cells or other cells as a result of cancer and have been shown to be attractive targets for early diagnosis, monitoring therapy response, detecting recurrence, or following up prognosis of cancer [11]. Proteins are expressed after DNA and RNA have been transcribed and translated and they subsequently perform the desired functions. Due to protein biomarkers being more prevalent than RNA or DNA, they are crucial for the clinical diagnosis of diseases [12]. Due to their poor stability and low abundance in bodily fluids, their precise detection is often influenced by the complexity of the environment. In order to design protein biosensors, sensitivity, specificity, and accuracy are key parameters to be considered. So far, 19 protein cancer markers have been approved by the U.S. Food and Drug Administration (FDA). Of these, 11 markers are found in blood, 5 in tissue, and 3 in urine [13]. Of all of these, prostate specific antigen (PSA), carcinoembryonic antigen (CEA), Epidermal growth factor receptor (EGFR), Human epidermal growth factor receptor (HER/ErbB) and Carbohydrate antigen (CA) and its types are considered to be very important. The list of protein biomarkers along with its related cancer type is given in Table 1.

### 3.1. Electrochemical Detection

**Figure 1:**
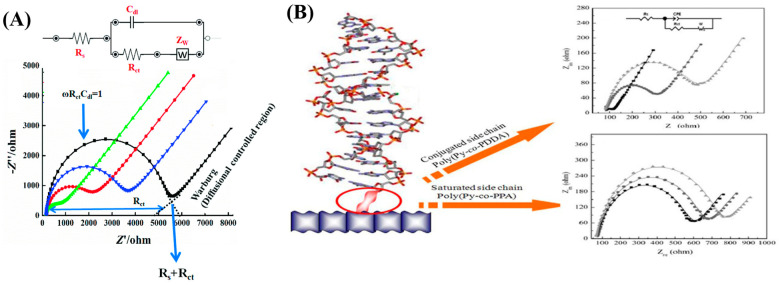
(**A**) Equivalent circuit of the EIS. Reproduced with permission from Ref. [14] and (**B**) Nyquist plots for electrochemical impedance measurements for poly(Py-co-PPDA) with and without immobilization of probe DNA. Reproduced with permission from Ref. [15].

Over the last few decades, electrochemical biosensors are widely employed due to their ability to precisely detect protein biomarkers with the goal of improving cancer diagnosis by simple operation, point-of-care and real-time analysis [11]. Electrochemical impedance spectroscopy (EIS) is widely used in immunosensor and aptasensor for the detection of cancer biomarker. Figure 1A,B are the representative equivalent circuit and biomarker detection protocol employed in impedance sensor towards cancer biomarker detection.

**Figure 2:**
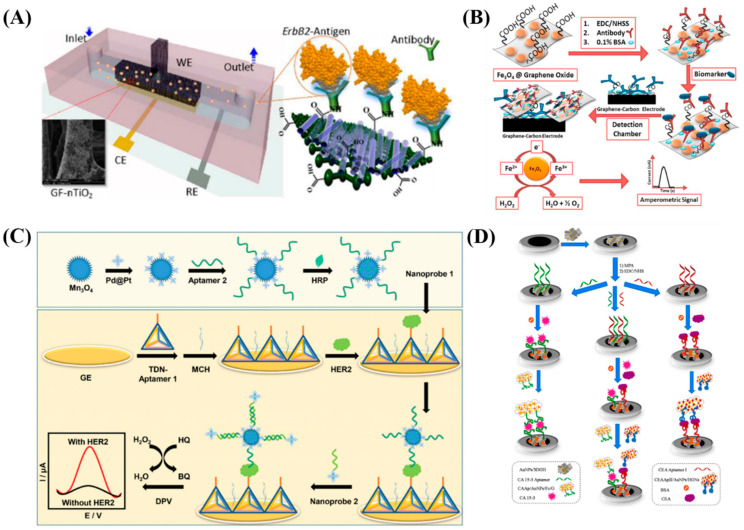
(**A**) Schematic representation of the graphene foam (GF) modified carbon-doped titanium dioxide nanofibers microfluidic immunosensor. Re-used with permission from Ref. [16]. (Copyright 2016 American Chemical Society) (**B**) Overall representation of the Fe_3_O_4_@GO work. Re-used with permission from Ref. [17]. (Copyright, 2017 Elsevier, reproduced with permission from Elsevier Ltd.) (**C**) Scheme of the design of electrochemical Mn_3_O_4_/Pd@Pt/HRP dual-aptamer biosensor. Re-used with permission from Ref. [18] (Copyright, 2019 Royal society of chemistry, reproduced with permission from Royal society of chemistry Ltd.) and (**D**) schematic steps involved in the preparation of AuNPs/3DGH sandwich–type aptasensor. Re-used with permission from Ref. [20]. (Copyright, 2021 Elsevier, reproduced with permission from Elsevier Ltd.).

A label-free microfluidic immunosensor designed using graphene foam (GF) modified carbon-doped titanium dioxide nanofibers exhibited excellent selectivity and sensitivity towards the detection of epidermal growth factor receptor 2 (EGFR2 or ErbB2) proteins. This sensor was subjected to electrochemical impedance spectroscopy and differential pulse voltammetry to quantify breast cancer biomarkers. These techniques revealed high sensitivities of 0.585 µA µM^−1^ cm^−2^ and 43.7 kΩµM^−1^ cm^−2^ in a wide concentration range of from 1.0 fM to 0.1 µM and 0.1 pM to 0.1 µM. Even in the presence of identical members of the EGFR family of receptor tyrosine kinases, such as ErbB3 and ErbB4, the use of the specific recognition element (i.e., anti-ErbB2) offered great specificity indicating the efficacy. The schematic of a microfluidic immunosensor is shown in Figure 2A [16]. PSA, also known as human kallikrein 3 (hK3 or KLK3), is one of the earliest discovered serological prostate cancer (PC) indicators widely used in clinical interpretation of prostate cancer. Since PSA levels exceeding 4.0 ng/mL are typically regarded as abnormal, this value is the internationally accepted cut-off value for PC formation. Fe_3_O_4_ nanoparticles modified graphene oxide (GO) nanosheets were designed by Mohamed Sharafeldin et al., as an approach for the ultrasensitive mediator-free electrochemical detection of PC biomarker proteins. The biomarker enriched Fe_3_O_4_@GO particles are selectively captured by screen-printed carbon electrodes covered by electrochemically reduced graphene oxide (ERGO) and a second set of antibodies, which then catalyze hydrogen peroxide reduction to detect PSA and PSMA. The enzyme-linked immunosorbent tests and patient serum assays exhibited good correlation thereby demonstrating accuracy (ELISA) with excellent detection limits (LOD) of 15 fg/mL for PSA and 4.8 fg/mL for PSMA in serum. The scheme of this work is depicted in Figure 2B [17].

The overexpression of HER/ErbB metabolite family is generally linked to a variety of cancers. The first growth factor receptor from the HER family connected to oncoproteins was EGFR (HER1/ErbB1). Its over-expression has been linked to a number of malignancies, including non-small cell lung cancer, breast, ovarian, prostate, pancreatic, renal, head and neck, colorectal cancer, a bad prognosis and the emergence of aggressive disease stages [13]. The detection of the breast cancer cell biomarker HER2 using an electrochemical aptasensor of the Mn_3_O_4_/Pd@Pt/HRP sandwich type has been reported. Herein, the tetrahedral DNA nanostructures (TDNs)-aptamer served as recognition probes for the aptasensor, and flower-like nanozymes and horseradish peroxidase (HRP) served as signal nanoprobes. The TDN-aptamer 1 was adsorbed on the gold electrode surface during the biosensor’s construction as a biorecognition component for detecting the biomarker HER2.The built-in biosensor exhibits excellent repeatability, superior stability, a wide linear range, low LOD, high selectivity, and a high degree of precision. The design protocol of the electrochemical dual-aptamer biosensor is shown in Figure 2C [18]. With 25% of all cases and 15% of all cancer-related fatalities, breast cancer (BC) takes center stage as the most frequently diagnosed cancer type worldwide [19]. A sandwich-type electrochemical aptasensor was developed using a gold/graphene hydrogel (AuNPs/3DGH) nanocomposite for the simultaneous detection of two key breast cancer biomarkers, carcinoembryonic antigen (CEA) and cancer antigen 15-3 (CA 15-3). In this work, hemin and ferrocene served as electrochemical signal for the dual biomarker detection and as redox probes for CEA and CA 15-3, respectively. The aptamers CEA and CA 15-3 were immobilized on AuNPs/3DGH-modified electrode’s surface. The duplexed aptasensor’s surface exhibited 11.2 pg/mL^−1^ and 11.2 × 10^−2^ U/mL detection limits for CEA and CA, respectively. The scheme of the different steps involved in the preparation of the sandwich–type aptasensor is shown in Figure 2D [20]. Carboxyl terminated graphene nanosheets (CGS) are used as a “carrier” for both indicator materials and antibodies in a sandwich immunosensing protocol developed by Chen et al., for the simultaneous detection of carcinoembryonic antigen (CEA) and alpha-fetoprotein (AFP). The glassy carbon electrode surface (GCE) was modified with chitosan and gold nanoparticles, followed by the immobilization of a capture antibody. In this work, the detection limits were found to be 0.05 ng/mL for AFP and 0.1 ng/mL for CEA using non-amplification techniques [21].

Despite the fact that cytokines have traditionally been thought of as cancer biomarkers, a few other glycoproteins, most commonly CEA and tissue inhibitor of metalloproteinase 1 (TIMP1), are involved in cellular adhesion that is frequently produced in gastrointestinal tissue and their abnormal levels have been found in serum of lung, colorectal and breast cancer [22]. Recently, the development of paper-based electrochemical biosensors attracted researcher’s attention despite the fact that numerous conventional and non-traditional methods have been developed over the years for the detection and measurement of cytokines and cancer biomarkers. This is because of their unique characteristics such as device portability, specificity, sensitivity, ease of use, and affordability [23]. A novel semiconducting nitrogen-rich tetrazine polymer was used as an immobilizing matrix for the anti-CEA due to its exceptional capacity to form potent H-bonding contacts with the antibody. Joshi et al., reported on the end-to-end construction of this novel and straightforward technology. In order to facilitate binding, the CEA was dropped onto the anti-CEA/PhPTz/rGO devices at ambient circumstances. The change in current passing through the sensors was then monitored. When the devices were tested for a wide range of CEA concentrations (0.25 pg/mL–800 ng/mL), a response of 2.75–33.7 μA was recorded. The electronics and algorithm employed were found to have excellent prediction accuracy [23]. Li et al., developed novel electrochemical immunosensor for the sensitive and label-free detection of CA 15-3. With a highly conductive electrode made of N-doped graphene sheets, this special immunosensor demonstrated a marked increase in electron transfer and exceptional sensitivity towards the detection of CA 15-3. With a narrow linear range of 0.1–20 U/mL and a low detection limit of 0.012 U/mL, this new immunosensor performed effectively. The use of highly conductive graphene restricts the need for labels and is more straightforward than conventional immunosensors, which typically involve intricate label processing and time-consuming separations. The method created for this immunosensor offers a potential strategy for applications in clinical study and diagnostics [24].

### 3.2. Optical Based Detection

**Figure 3:**
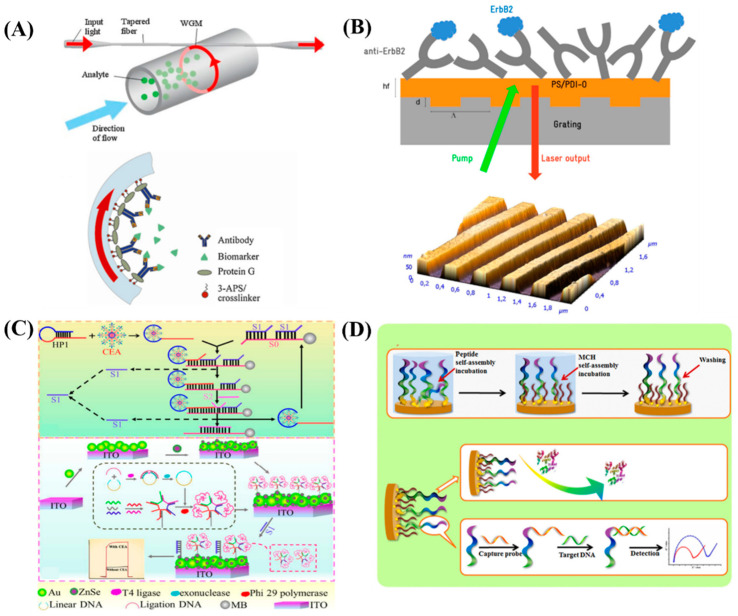
(**A**) Schematic representation of the OFRR sensor. Re-used with permission from Ref. [25] (Copyright, 2010 Elsevier, reproduced with permission from Elsevier Ltd.). (**B**) Scheme of the DFB laser sensor. Re-used with permission from Ref. [26]. (Copyright, 2016 Elsevier, reproduced with permission from Elsevier Ltd.) (**C**) Schematic of the PEC biosensor for CEA determination. Re-used with permission from Ref. [27]. (Copyright, 2016 Elsevier, reproduced with permission from Elsevier Ltd.) and (**D**) Schematic illustration of Peptide SAM formation, fabrication and sensing procedures. Re-used with permission from Ref. [28]. (Copyright, 2017 Elsevier, reproduced with permission from Elsevier Ltd.).

In order to overcome the pitfall of conventional biomarker sensing approaches, Gohring et al. [25] demonstrated a ring resonator sensing technology. Herein, a brand-new, label-free opto-fluidic ring resonator (OFRR) for finding the HER2 extra-cellular domain breast cancer biomarker in samples of human serum was reported. The OFRR employ optical ring resonator sensing technologies and microfluidics to offer quick label-free detection on a tiny, affordable platform. According to the results, the OFRR can identify HER2 in serum between 13 and 100 ng/mL that are medically significant in <30 min. A polymer can also be used to pack this gadget to boost its mechanical toughness. It offers label-free detection thereby allows major simplification of the entire detection protocol. The scheme of the OFRR sensor is shown in Figure 3A. Retolaza et al. [26] developed a vertically emitting organic distributed feedback (DFB) laser to identify the ErbB2 protein. This DFB laser was made up of a second-order one-dimensional grating that was created on fused silica using thermal-nanoimprint lithography and reactive ion etching procedures, which was covered in a polystyrene (PS) layer containing a perylenediimide laser dye. Upon functionalizing PS with anti-ErbB2 monoclonal antibodies, the system’s specificity to the ErbB2 protein biomarker is established. It has been discovered that the tumor necrosis factor alpha (TNF) and bovine serum albumin (BSA) proteins will notinteract with the ErbB2 protein thereby proving its selectivity. The schematic of DFB laser sensor is shown in Figure 3B. A photoelectrochemical (PEC) biosensing platform was created using a 3D DNA nanostructure that was self-assembled by base complementary pairing in a few minutes, rolling circle amplification (RCA), and multiple enzyme-free amplification strategies. This platform enables the ultrasensitive detection of CEA. The high PEC signal of the ZnSe QDs and the high amplification effectiveness of the multiple SDA reaction are responsible for the good performance of the proposed biosensor. The 3D DNA nanospheres on the electrode effectively decreased the photocurrent signal from ZnSe QDs, turning it from “on” to “off” state. Later, CEA-induced recycling amplification produced a large number of S1 to replace the DNA nanospheres from the electrode, turning the PEC signal back into an “on” state to enable sensitive CEA assay. The suggested PEC biosensor demonstrated an outstanding performance with a broad linear range of 1.0 fg/mL to 10 ng/mL with a low detection limit of 0.12 fg/mL for CEA and used for the biomarker analysis in real blood samples. The schematic diagram of this PEC biosensor for CEA determination is shown in Figure 3C [27]. A zwitterionic peptide self-assembled monolayer (SAM) support that serves as the low fouling substrate was used to construct a highly sensitive and selective label free electrochemical DNA hybridization biosensor for the breast cancer marker BRCA1. Due to its exceptional hydrophilicity and charge neutrality, the peptide SAM supporting interface was demonstrated through EIS experiments to be able to prevent the nonspecific protein adsorption onto the sensing interface even in 10% (*v*/*v*) human serum. A linear range of 1.0 fM to 10.0 pM and a detection limit of 0.3 fM were attained by the sensitive impedance-based assay for BRCA1-related sequence, which was also effective to identify DNA mismatches [28]. The schematic illustration of peptide SAM formation, design and sensing procedures are shown in Figure 3D. The serological biomarker panel of glypican-3 (GPC3), dickkopf-1 (DKK1), and AFP was proposed by Cheng and Fu [29] for the detection of liver cancer. The simultaneous detection of the biomarker panel by fluorescence was achieved by utilizing mixed reference samples comprising of human recombinant GPC3, DKK1, and AFP antigens as a proof-of-concept. Their method of simultaneous detection exhibited a linear range of 0.625–2.5 ng/mL for all the biomarker panel which justifies additional clinical testing to confirm that the biomarker panel can be accurately and simultaneously determined in human serum samples. The unclad OF-SPR approach involved an equipment that is both affordable and easy to readout. Through OF-SPR optrodes, identification of breast cancer HER2 biomarkers was simple. Multiple assays verified the findings, and by implementing biosensing with a direct and indirect strategy focused on HER2 proteins, the dataset was enhanced. Target biomarkers were specifically identified at 1 g/mL using a label-free method, while HER2-antibodies produced a 100× improvement in the threshold and thereby it reaches 10 ng/mL (86 pM) after amplification [30].

A variety of new artificial enzymes were developed due to the rapid progress in nanotechnology and biotechnology. Nanozymes proved to be very effective in clinical medicine, biopharmaceuticals, environmental monitoring, and many other sectors as promising natural enzyme mimics [31]. The most recent advancements include study of nanozymes based on metals and metal oxides in the analytical, antibacterial, inflammation-relieving and cancer therapeutic fields [32]. Iron oxide nanoparticles (IONPs) have been used extensively to detect and destroy cancer cells. When exposed to a magnetic field, these inorganic particles may become magnetized and adhere to cancer cells. Utilizing an external magnetic field, IONPs can be conjugated with anticancer medications, antibodies, nucleotides, enzymes, and proteins to deliver them to the precise target site [33]. Recently, the use of bioactive glasses in cancer therapy has grown extremely in terms of fighting cancer cells while regenerating lost bone tissue. Therefore, a number of Fe doped 45S5-based mesoporous bioactive glasses for prospective uses in cancer therapy based on initiating Fenton’s reactions were proposed and promising results were obtained thereby confirming the usefulness of Fe based materials in cancer therapy [34]. The presence of tunable band gap, low cost, wide specific area, and simplicity of manufacture, metal-oxide nanoparticles (MONs) have attracted attention towards the development of flexible/wearable sensors. Flexible MONs nanocomposites and polymers including PVDF, PANI, and PVA are frequently employed. When MONs were the major sensing components, the polymers in nanocomposites serve primarily as MON anchors to avoid mechanical damage, such as thin film cracking. In this situation, a sufficient MON concentration was essential to build MON percolation networks that provide electrical connection. MONs have also been used as different types of additives to improve the performance of electrically functional polymers such as PVDF and PANI. Although redox reactions and photoelectric phenomena can be better detected by MON-based flexible sensors, advancements in MON-based thermistors and mechanical sensors is expected to open up opportunities for newer technology [35].

## 4. Nucleic Acid Based Biomarkers

### 4.1. DNA Based Nucleic Acid Biomarkers

**Figure 4:**
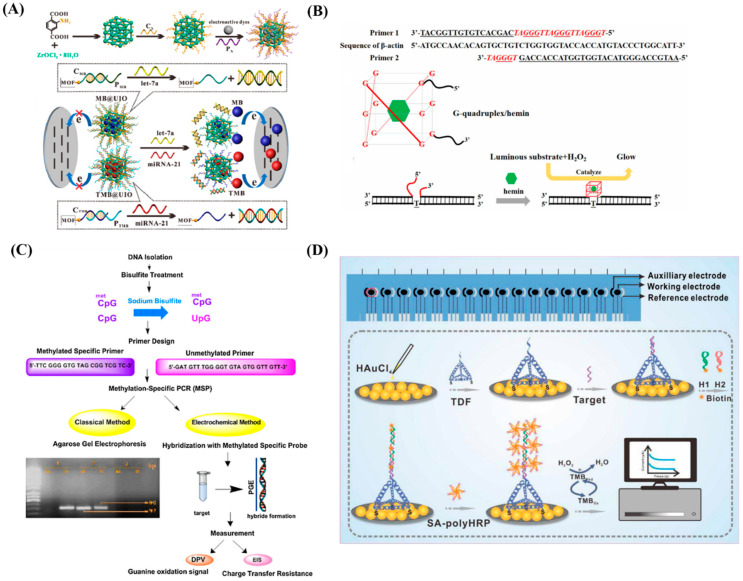
(**A**) Schematic representing the design and working principle of the MOF based biosensor. Re-used with permission from Ref. [39] (**B**) Overall mechanism of DNA G-quadruplex/hemin enzyme. Re-used with permission from Ref. [42] (Copyright, 2021 Elsevier, reproduced with permission from Elsevier Ltd.). (**C**) Schematic represents the detection of GSTP1 hypermethylation by electrochemical and electrophoretic methods. Re-used with permission from Ref. [45]. (Copyright, 2012 Elsevier, reproduced with permission from Elsevier Ltd.) and (**D**) Schematic representation of the E-cfDNA sensor Re-used with permission from Ref. [46].

The first description of cell-free nucleic acid (cfNA) in human blood was during 1948 by Mandel and Métais [36]. Cell-free DNA (cfDNA) concentrations in breast cancer patients’ serum range from 0 to 2 ng/mL. It was possible to evaluate fluctuations in the amount, depending on the stage of the disease and the patients’ responses to treatment. Microsatellite changes on cell-free DNA (cfDNA) were first observed in cancer patients in 1996. The death and necrosis of cancer cells in the tumor microenvironment are assumed to be associated to the release of nucleic acids into the blood and secretion has also been proposed as a potential source of cfDNA. The blood-circulating tumor cells and micrometastatic deposits seen in organs such as liver and bone marrow may also contribute to the release of cfNA. The blood and lymphatic circulation’s clearance, degradation and other physiological filtering processes also have an impact on the amount of cfNA. One of the key elements in the turnover of cfNA may be the nuclease activity in blood. Given that they may be more informative, specific, and accurate than protein biomarkers, cfNAs are considered as effective blood cancer biomarkers. Currently, accurate tumor staging, early diagnosis, and treatment monitoring are key components of effective cancer management. The “gold standard” of diagnosis is the histological analysis of tumor tissues acquired from biopsies as well as blood samples. However, most studies perform these analyses only once [37,38]. For the simultaneous detection of the tumor biomarkers let-7a and miRNA-21, Chang and his team proposed a dsDNA-functionalized MOF based homogenous label-free electrochemical approach, where dsDNA is used to cap MOFs. Through in situ adsorption and a nucleic acid hybridization procedure, the functionalized MOFs (dsDNA-capped MOFs) were created, which were loaded with electroactive dyes and covered by double-stranded DNA. This biosensor exhibited excellent performance with high sensitivity, selectivity and LODs of 3.6 fM for let-7a and 8.2 fM for miRNA-21, respectively. Such a performance can be attributed to the UIO-66-NH_2′_s highly porous structure and the superior gating effect of dsDNA over ssDNA. The schematic representation of the design, protocol of this biosensor is shown in Figure 4A [39]. Hao et al. [40] investigated the concentrations and integrity index of circulating cf-DNA in serum for clinical usage in colorectal cancer (CRC) patients’ diagnosis and progression monitoring. The novel magnetic bead approach was found to be rapid and high throughput. Moreover, it can create high yields of DNA with minimal error, ensuring a high degree of reproducibility. Mathios et al. [41] used a machine learning model for detecting tumor-derived cfDNA using genome-wide assessments of cfDNA fragmentation in 385 healthy individuals and 46 lung cancer patients. The detection of 94% patients with cancer across stages and subtypes, including 91% of stage I/II and 96% of stage III/IV, was achieved with 80% specificity by combining fragmentation features, clinical risk factors, and CEA levels followed by CT imaging. Individuals with small cell lung cancer were accurately differentiated from those with non-small cell lung cancer. Using a chemiluminescence DNA biosensor based on DNA G-quadruplex/hemin enzyme, Ying et al. [42] developed a novel technique for diagnosing GBC through quantification of circulating free DNA in serum samples from 228 participants. With the insertion of enough probes, the expression of the actin gene was assessed to evaluate the serum circulating free DNA levels and discovered that the concentration of circulating free DNA was much higher in the GBC group than in the healthy group. The findings demonstrated that the chemiluminescence DNA biosensor system’s diagnostic utility was virtually on par with that of qPCR, andthis technique clearly discriminated patients with GBC from healthy donors and patients with cholecystitis. The overall mechanism is shown in Figure 4B. Seminal plasma cfDNA from patients with prostate cancer has been quantified and its size distribution was evaluated by fluorometric quantification and electrophoretic analysis. The outcomes revealed three key facts. First, human seminal fluid is an important biologic fluid for the discovery of novel oncological biomarkers. Second, seminal plasma cfDNA from prostate cancer patients is significantly more concentrated than that of from age-matched healthy individuals. Third, fluorometric and electrophoretic assessments allow a reliable quantification and qualification of seminal plasma cfDNA, which could be used to identify novel oncological biomarkers [43]. N-doped MGA/GNS, or graphene aerogels doped with nitrogen, are used as an electrochemical sensing platform for the detection of double-stranded DNA (dsDNA). The integration not only results in an ultrafast DNA electron and charge transfer but also brings about a considerable synergy between N-doped MGA, GNS, and dsDNA. The N-doped MGA offered significantly higher electrochemical performance with a detection limit of 3.9 × 10^−22^ g/mL (S/N = 3), the DPV signal increases linearly with the concentration of dsDNA in the range of 1.0 × 10^−21^ g/mL to 1.0 × 10^−16^ g/mL [44]. A single-use carbon graphite-based label-free electrochemical genosensor for the detection of glutathione S-transferase P1 (GSTP1) hypermethylation through a hybridization event with DNA oligonucleotides has been disclosed. 2.92 pmol of the target sequence in a 100-L reaction volumeto be the limit of detection (S/N = 3). Compared to traditional optical and agarose gel electrophoresis techniques, the genosensor assay is less expensive. The schematicdetection of GSTP1 hypermethylation by electrochemical and electrophoretic methods is shown in Figure 4C [45]. A breast cancer susceptibility gene 1 (BRCA-1) detection system based on an ultrasensitive cfDNA electrochemical biosensor called tetrahedral DNA framework (TDF)-modified gold nanoparticles (AuNPs) was reported. Three different types of TDFs were programmed to control the number of base pairs on each DNA framework: TDFs with 26 base pairs, TDFs with 17 base pairs, and TDFs with 7 base pairs. The E-cfDNA sensor has the potential to be used in clinical research because it has an ultra-low detection limit of 1 aM and a linear range from 1 aM to 1 pM by TDF-26 and BRCA-1 in mock serum samples. The schematic of E-cfDNA sensor is shown in Figure 4D [46].

### 4.2. RNA Based Nucleic Acid Biomarkers

**Figure 5:**
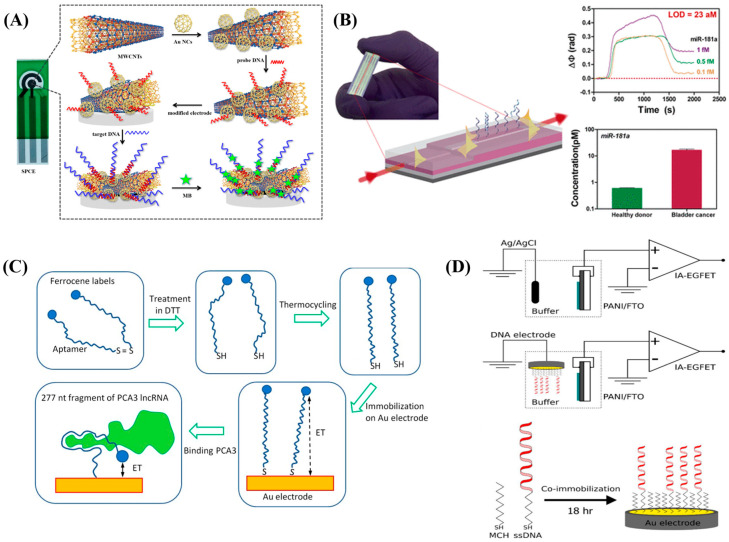
(**A**) Schematic representation of the SPCE electrochemical DNA biosensor. Re-used with permission from Ref. [50]. (**B**) Design and working principle of a BiMW sensor chipRe-used with permission from Ref. [51]. (Copyright 2022 American Chemical Society) (**C**) step–by–step design of aptasensor. Re-used with permission from Ref. [52] and (**D**) schematic representation of a potentiometric IA-EGFET sensor. Re-used with permission from Ref. [53]. (Copyright, 2021 Elsevier, reproduced with permission from Elsevier Ltd.).

RNAs are widely studied for transcriptional and post-transcriptional control in addition to their role as carriers of genetic information [47]. RNAs are unstable under alkaline environment. However, even at very low concentrations, they are easy to detect and quantify. RNA is more sensitive and selective than protein biomarkers. Using PCR, RNA sequence traces can be amplified and thus RNA can be detected with specificity and sensitivity. Similarly, it was demonstrated that the breast cancer patients’ serum can also include cell-free RNA (cfRNA), namely telomerase mRNA. MicroRNA (miRNA) is a short, single-stranded, non-coding RNA that has 18–25 nucleotides. MiRNA expression levels and the beginning and progression of diseases such as cancer, diabetes, and heart disease are closely connected [47,48]. On-chip, straightforward enzymatic in situ synthesis of ssRNA microarrays for the identification of proteins in a microfluidic format surface plasmon resonance imaging of biomarkers was reported. Multiple RNA aptamers could be produced simultaneously without further purification, no need for separate reaction compartments or spotting procedures and without the need of scaling with the number of initial distinct aptamer sequences [49]. As diagnostic biomarkers for the detection of non-small cell lung cancer and to track disease progression, long non-coding RNAs (lncRNAs) have been proposed. Based on a gold nanocage and a carbon electrode adorned with a screen-printed multi-walled carbon nanotube (Au NCs/MWCNT-NH_2_), a unique effective and ultrasensitive electrochemical biosensor was designed (SPCE). This SPCE Au NCs/MWCNT-NH_2_lncRNA biosensor demonstrated a wide linear range (10^−7^–10^−14^ M) and low limit of detection limit (42.8 fM), along with adequate selectivity and stability. The performance can be attributed to its high surface area, superior conductivity and excellent biocompatibility. This biosensor outperformed other conventional RT-PCR results in terms of acceptable stability, good selectivity, ease of operation, rapid analysis and cost. The schematic representation of the SPCE electrochemical biosensor is shown in Figure 5A [50]. In order to detect microRNAs in complex media with extreme sensitivity, Huertas et al. [51] demonstrated a nanophotonic biosensor based on interferometric bimodal nanowaveguides (BiMW) with possible multiplexing possibilities. In a broad dynamic range of concentrations, discrimination between the many homologous and pre-miRNA of miR-181a has been accomplished (aM–pM). Without the need for pre-sample preparation steps, the proposed BiMW biosensor was employed for the first time to directly detect and quantify miR-181a at aM concentration (LOD = 23 aM) in the urine samples of bladder cancer patients. The working principle of a BiMW sensor chip is shown in Figure 5B. Prostate cancer biomarker, prostate cancer antigen 3 (PCA3) was electrochemically detected in vitro in a direct test using a specific RNA aptamer labelled with a redox group (ferrocene), anchored on a screen-printed gold electrode surface. EIS and CV both exhibited detection in the 1 g/mL–0.1 ng/mL range. Herein, the EIS exhibited a LOD value of 0.03 ng/mL and CV exhibited a LOD of 0.04–0.09 ng/mL. The affinity constant for PCA3 to bind with aptamer was found to be 4 × 10^−10^ M, indicating a highly specific binding response similar to antigen-to-antibody interactions. The step-by-step design of aptasensor is shown in Figure 5C [52]. Similarly, DNA/RNA (DNA-EGFET) biosensor made of polyaniline (PANI)-based extended-gate field effect transistor functionalized Au reference electrode was reported. The transduction system is based on an electrodeposited PANI thin film as the sensing platform. Variations in the hybridized single strand DNA’s net surface charge caused changes in the FET system’s output voltage, which might be connected to quantify the complimentary DNA that is being detected. Fast reaction time, linear response in the detection range of 1 pmol/L to 1 mol/L, and LOD of 9.77 pmol/L were all the interesting features of this DNA-EGFET biosensor. The schematic representation of a potentiometric IA-EGFET sensor is shown in Figure 5D [53].

For the sensitive and precise detection of miRNA-21, a facile electrogenerated chemiluminescence biosensor based on carbon nanodots was developed. On the electrode surface, the target miRNA-21 and the probe underwent a hybridization reaction that was detected by CNDs by enhancing the [Ru(bpy)_3_]^2+^/DNA electrochemiluminescence signal. The biosensor exhibited a linear response to miRNA-21 concentrations up to 100.0 pM with a detection limit of 0.721 fM. The technique offered a rapid response time and did not require labelling steps. Without RNA extraction or amplification beforehand, it was successfully employed to detect miRNA-21 in the serum samples of heart failure patients [54]. So far, potential applications of miRNA in ovarian cancer [55], the role of peptide nucleic acids [56], optical materials [57] and electrochemical sensors on cancer diagnosis [57] were reported.

### 4.3. CRISPR

Diagnostics based on clustered regularly interspaced short palindromic repeats (CRISPR) offer the potential to use single-nucleotide specificity, which is essential for identifying mutations that exhibits resistance to antibiotics or antiviral medications [58]. A crucial component of a microbial adaptive immune system, CRISPR systems identify foreign nucleic acids based on their sequence and then remove them via endonuclease activity linked to the CRISPR-associated (Cas) enzyme [59]. According to the reports, the CRISPR/Cas9 system is an RNA-guided DNA-targeting endonuclease that operates in a sequence-specific manner. The important phases of the CRISPR/Cas9-based genome editing mechanism are as follows: DNA acquisition from the invading phage particle (adaptation), CRISPR/Cas assembly formation, target DNA annihilation or interference, and the insertion of the desired gene sequence. Only 20 nucleotides in the guide RNA must be changed in the CRISPR/Cas system to retarget the Cas protein than 500–1500 base pairs for ZFNs and TALENs. Furthermore, it is simple, easy to prepare, and more versatile than other gene editing platforms. The CRISPR/Cas system can target multiple target sites simultaneously in the same cell by using multiple guide RNAs [60]. According to the quantity and sequence of cas genes linked to CRISPR arrays, CRISPR-Cas systems are divided into six types (I–VI) and two classes (Class 1: types I–IV; Class 2: types II–V and VI). For the rapid therapeutic applications of these nucleases, it is crucial to detect and minimize off-target effects caused by CRISPR-Cas systems along with combining extramolecular tools to the CRISPR arsenal. Multiple stages are necessary for CRISPR-mediated genome editing, including Cas9 target recognition, binding and cleavage. Several cutting-edge techniques have been created in recent years to investigate target recognition and to describe the genome-wide specificities of CRISPR-Cas9 [58].

The potential of CRISPR technology in biosensing was reported by Chen et al. [61] who further summarized its application tactics in molecular diagnostics. Cas9, Cas12, and Cas13, along with their respective subtypes, are currently the three Cas enzyme types that are most widely employed. The key for signal amplification is provided by Cas9′s cis-cleavage capability, Cas12 and Cas13′s trans-cleavage capability, with the hope that additional Cas proteins will be discovered or modified in the future that are expected to possess more advantages [61]. Based on their mode of action, CRISPR/Cas-based biosensors for protein detection can be classified under three categories: antibody-assisted CRISPR/Cas-based protein detection, aptamer-assisted CRISPR/Cas-based protein detection, and other CRISPR/Cas-based approaches for protein detection. Aptamers are used as signal recognition components in CRISPR-based biosensors for protein detection due to their superior molecular properties and integration. Aptamers work well with CRISPR/Cas systems for protein recognition, converting protein signals to nucleic acid signals with activated Cas and signal output. Furthermore, as SELEX technology is progressing quickly, more and more protein aptamers will be found, thus, expanding the use of aptamer-assisted CRISPR/Cas biosensors for protein detection [62]. Genomes of cancer cells contain a variety of genetic alterations that build up from inherited and acquired mutations and are brought by repeated clonal expansions. In gene loss-of-function research, large-scale screening utilized CRISPR/Cas9 knockout libraries was frequently employed. Another significant CRISPR/Cas9-based technique, CRISPRi (CRISPR inhibition), was created for loss-of-function testing in cancer research. CRISPRi can accurately interfere with any lncRNA gene since it can operate only within a narrow (1 kb) radius of the target transcription start site (TSS) and dCas9 only blocks 23 bp of the targeted sequence. Collectively, CRISPRa library screening offers more sophisticated methods to study tumors and serve as an useful manual for the clinical treatment of cancer [63].

**Figure 6:**
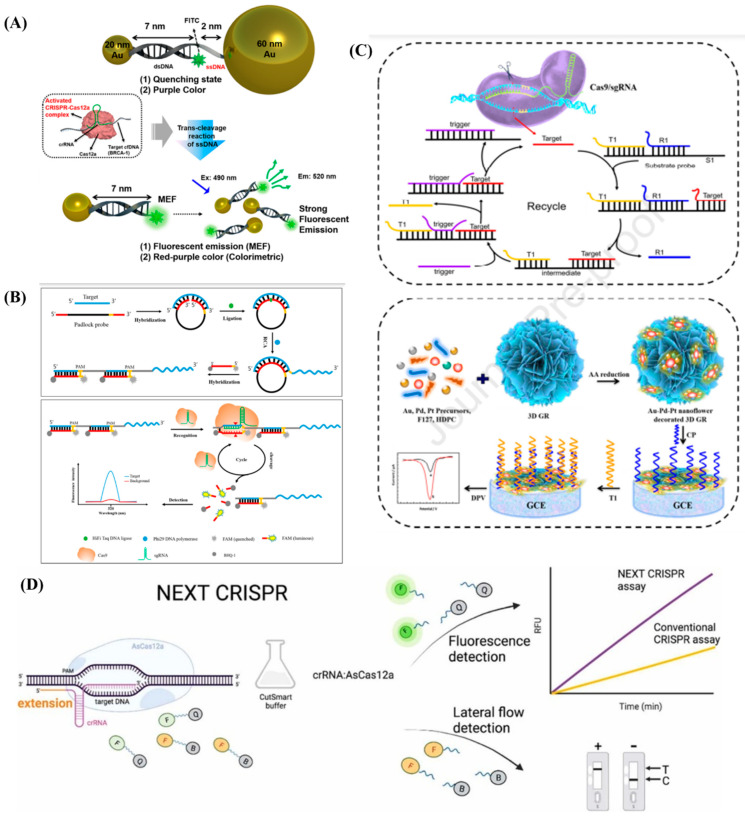
(**A**) Schematic illustration of cfDNA detection. Re-used with permission from Ref. [64]. (Copyright 2021, American Chemical Society). (**B**) Overall reaction mechanism of RACE. Re-used with permission from Ref. [65]. (Copyright 2020, American Chemical Society). (**C**) Schematic illustration the operation of the 119 3D GR/AuPtPd nanoflower biosensor–based CRISPR/Cas9–triggered ESDR. Re-used with permission from Ref. [66]. (Copyright, 2021 Elsevier, reproduced with permission from Elsevier Ltd.) and (**D**) Schematic representation of NEXT CRISPR biosensing platform. Re-used with permission from Ref. [67] (Copyright, 2022 Elsevier, reproduced with permission from Elsevier Ltd.).

Although fluorescent biosensors based on CRISPR-Cas have been designed, they too require an amplification step for detection. Upon employing DNA-functionalized Au nanoparticles (AuNP), Choi et al. [64] created the first CRISPR-Cas12a based nucleic acid amplification-free fluorescent biosensor to detect cfDNA. Metal enhanced fluorescence occurs with color changes from purple to red purple as a result of the target cfDNA activating the CRISPR-Cas12a complex and subsequent single-strand DNA (ssDNA) degradation between AuNP and fluorophore. Breast cancer gene-1 (BRCA-1) can be quickly and effectively identified with this approach. Other nucleic acid biomarkers, such as viral DNA can be measured using this quick and highly-selective sensor in a field-deployable and point-of-care testing (POCT) device. The schematic illustration of cfDNA detection is shown in Figure 6A. Upon combining the benefits of a clustered regularly interspaced short palindromic repeats/CRISPR associated nucleases (CRISPR/Cas) system and rolling circular amplification (RCA) techniques, Wang et al. [65] developed a highly specific nucleic acid detection platform for the simultaneous quantification of several EV-derived miRNAs at constant temperature. Due to the dual-specific identification from both padlock probe-mediated ligation and protospacer adjacent motif (PAM)-triggered cleavage, the suggested technique specifically displayed single-base resolution. The robustness of the suggested RCA-assisted CRISPR/Cas9 cleavage (RACE) and reverse transcription quantitative polymerase chain reaction (RT-qPCR) in determining the abundance of EV-derived miRNAs from both clinical lung cancer patients and cultured cancer cells was confirmed by the high consistency of the two methods. This revealed the method’s ability in screening, diagnosis and prognosis of various diseases. The overall reaction mechanism of RACE is depicted in Figure 6B. For the efficient detection of ctDNA, Chen et al. [66] designed a novel 3D GR/AuPtPd nanoflower sensing platform based on the entropy-driven strand displacement reaction (ESDR) that was caused by CRISPR/Cas9 cleavage. This technique enabled the detection of low quantities of ctDNA since ESDR amplification necessitates intricate operating procedures and reaction conditions. Thistechnique is highly specific for distinguishing single-nucleotide mismatches and amplification efficiency by fusing the benefits of the rapid amplification kinetics of entropy-driven strand displacement with those of the site-specific cleavage by Cas9/sgRNA. In the tests using human serum, the 3D GR/AuPtPd nanoflower-based electrochemical biosensor exhibited excellent specificity. As a result, this ground-breaking technique offers a fresh perspective on effective ctDNA detection for applications in therapeutic and diagnostic settings. The schematic illustrating the basic operation of the 119 3D GR/AuPtPd nanoflower biosensor-based CRISPR/Cas9-triggered ESDR is shown in Figure 6C. The 5′ eNdEXTension CRISPR (termed “NEXT CRISPR”) biosensing platform was proposed for the rapid detection of nucleic acids with extreme sensitivity and specificity. The impact of extending the 5′ end of CRISPR RNA (crRNA) on CRISPR detection was also investigated. The NEXT CRISPR can be easily modified for both fluorescence detection and lateral flow strip reading, and it is compatible with recombinase polymerase amplification (RPA). They combined RPA/NEXT CRISPR with a lateral flow assay to create a NEXT CRISPR biosensing platform for human papillomavirus (HPV) 16 DNA detection with aM sensitivity within 30 min, enabling point-of-care testing. Furthermore, they achieved performance that was comparable to the traditional PCR method when they employed clinical swab samples to further clinically validate the NEXT CRISPR biosensing platform [67]. The protocol that followed was depicted in Figure 6D.

## 5. Extracellular Vesicles (EV) Based Biomarkers

Malignant tissue must be separated during conventional tissue biopsies used to diagnose cancer so that it may be analyzed using molecular and immunological techniques [68]. Highly sensitive cancer detection is made possible by direct sampling of damaged tissue, but doing so necessitates access to the exact localization of an affected region. Due to the hazards involved in taking tissue from specific locations, diagnostic biopsies for some malignancies are not possible, and it is rarely practical to take recurrent biopsies to assess the effectiveness of treatment. This method may result in false-negative results or inaccurate representations of the severity, course, or heterogeneity of the disease if the diagnostic sample being examined is not from the affected area or captures an unusual spot [68,69]. In this regard, extracellular vesicles (EVs) are heterogeneous vesicles produced by a number of mammalian cells, particularly cancer cells that are proliferating. A significant amount of EVs that can shuttle between parental and other cells are present in biofluids. EVs are now recognized as abundant and stable sources of bio-macromolecules such proteins, mRNA/miRNA, and DNA. Therefore, they can serve as cellular surrogates after initially being underappreciated as “cell dust” and a method to dispose of cellular components. These bio-macromolecules play a crucial role in the tumor microenvironment, participate in immune system adaptation and regulation, regulate pathological angiogenesis, including tumor angiogenesis, tumor growth, and metastasis. Therefore, EVs confirm substantial advantage in cancer surveillance. Tumor-associated vesicles can be employed as efficient surrogate biomarkers to determine the tumor type, stage, and underlying mutations, as well as to track the effectiveness of treatment [70]. EVs are widely distributed in blood and are also found in urine, saliva, and cerebrospinal fluid, among other bodily fluids. These bodily fluids can be collected non-invasively or without any serious intervention. There are numerous studies of liquid biopsy using EVs. Tumor cells are known to secrete more EVs than normal cells. In comparison to healthy adults, cancer patients serum had a higher concentration of EVs and aids in identifying cancer patients in the early and late stages [71]. Exosomes and microvesicles are difficult to separate because of overlaps in their physical characteristics and protein composition. However, EV subtypes are segregated by size, density and protein composition. Electrochemical biosensors combine biometric components (enzymes, proteins, antibodies, nucleic acids, cells, tissues, or receptors) that upon selective reactivity with a target analyte, produce signals that corresponds to the concentration of the analyte being examined. The electrochemical biosensors offer a significant advantage in the field of EV detection and may be useful for cancer screening, patient prognosis prediction and therapeutic applications. The use of DNA, lipids, and peptides as biomarkers for the detection of EVs is widely established. There are no reports on the electrochemical detection of EVs. Surface functionalization, sample matrix effects, and repeatability issues remain as a key challenge in this approach. Therefore, the probable breakthrough strategy will be the interfacial engineering research, pre-concentrating exosomes, design of stable electrodes based on nanomaterials [70].

**Figure 7:**
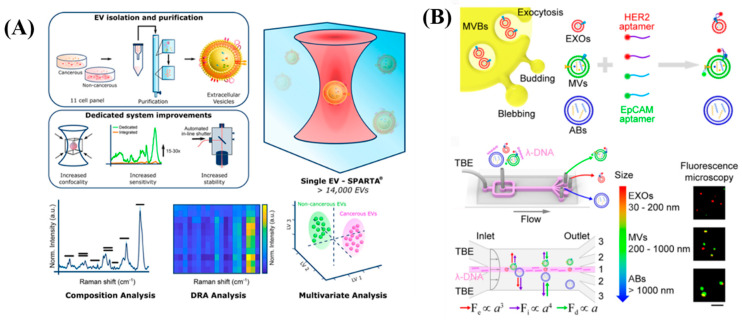
(**A**) Overall results of the SPARTA system. Re-used with permission from Ref. [74] and (**B**) Scheme of λ–DNA-mediated aptamer based analysis of individual EVs. Re–used with permission from Ref. [75]. (Copyright 2019 American Chemical Society).

Exosomes, a tiny fraction of EVs, are crucial in the modification of the tumor immunological milieu even before the onset and spread of cancer [72]. They are engaged in several physiological and pathological processes. Exosomes produced by host cells and tumor cells mediate their mutual regulation locally or remotely, affecting cancer treatments despite its effectiveness. As a result, circulating exosomes from tumors are regarded as non-invasive indicators for early tumor detection and diagnosis. Exosome-based therapeutics are also emerging as innovative and effective methods that might be used to inhibit tumor growth or improve anti-tumor immunity. A distinct miRNA expression pattern towards early stage liver fibrosis was found in whole plasma and its circulating vesicles by ECV-associated miRNAs [72,73]. A high-throughput, label-free extracellular vesicle analysis approach was realized with a view to cancer diagnosis and monitoring in a minimally invasive way. Herein, they used the single particle automated Raman trapping analysis (SPARTA) technology, which has a high degree of sensitivity and specificity (>95% for both) to distinguish cancer and non-cancer EVs. Accurate classification of EVs originating from multiple closely related breast cancer subtypes was revealed by comprehensive modelling that supports the use of the SPARTA-based approaches for detailed EV profiling [74]. The overall results of the SPARTA system is shown in Figure 7A. Liu et al. [75] demonstrated that isolated microvesicles are more effective than exosomes and apoptotic bodies in differentiating breast cell lines and Stage II breast cancer patients with varying immune histochemical expression of HER2. They used a machine learning algorithm to create an EV signature based on their size and marker expression. This research offered the first DNA-mediated method for classifying and identifying distinct EV subpopulations. By analyzing EV signatures, this platform made it possible to understand the heterogeneity of individual EVs and distinguish between breast cancer patients and cell lines. The schematic of λ-DNA-mediated aptamer-based analysis of individual EVs is shown in Figure 7B.

### 5.1. Microfluidics, Microarrays and FET Based EVs

**Figure 8:**
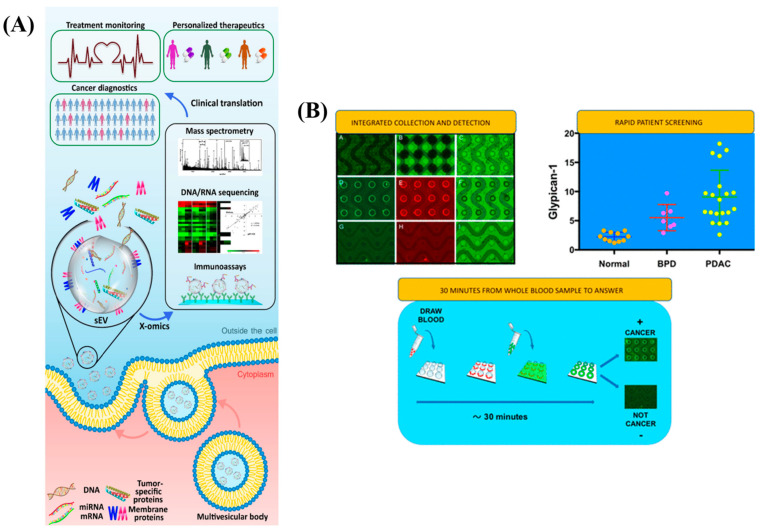
(**A**) Extracellular Vesicles as Next Generation Liquid Biopsy Biomarkers. Re-used with permission from Ref. [68]. (Copyright, 2021 Elsevier, reproduced with permission from Elsevier Ltd.). (**B**) ACE direct immunoassay procedure and its related results in whole blood and plasma/serum from pancreatic cancer patients. Re-used with permission from Ref. [80]. (Copyright 2018, American Chemical Society).

EVs exhibit surface indicators that can identify their cellular or tissue origin and contain a variety of payloads (DNA, RNA, protein, etc.) and have been shown to secrete at significantly higher rates from cancer cells and tumor tissue [68]. The schematic representation of EVs as Next Generation Liquid Biopsy Biomarkers is depicted in Figure 8A. EV manipulation techniques based on microfluidics have been developed in the last ten years whereas microfluidic technologies for separating CTCs have been created over the last 20 years based on various physical features or surface biomarkers of CTCs. Nanofabrication techniques have been employed significantly in size-dependent EV separation since EVs are smaller than CTCs. These tools have numerous scopes for cancer detection and therapy response monitoring. Surface biomarker-dependent and size-dependent strategies for EV separation based on microfluidics are the two groups. As of now, alternating current electrohydrodynamic microfluidic devices functionalized with anti-HER2 or anti-CD9 capture antibodies to separate EVs from cell culture medium and patient serum towards HER2, dual-patterned immunofiltration microfluidic chips with fluorescent dye conjugated anti-EpCAM to discriminate EpCAM, microfluidic devices on EVs collected anti-CD63 immunomagnetic beads for EpCAM and HER2 in breast cancer patient plasma, etc. were reported [71]. Two breast cancer microRNA biomarkers (microRNA-195 and microRNA-126) were rapidly detected using a small, DNA-FET biosensor-based integrated microfluidic system (IMS) in under 100 µL of plasma. The breast cancer biomarkers microRNA-195 and microRNA-126 were collected within 20 min by amine-modified cDNA-coated beads at rates of 85 and 94%, respectively. The IMS captured 84% of EVs using anti-CD63 beads from 100 µL of plasma within 4 h. Highly sensitive microRNA measurements over fM to 100 pM were made possible by a DNA-FET biosensor (gate width = 20 m) equipped with CMOS readout circuits and the entire diagnostic procedure can be completed in 5 h [76]. A new graphene oxide/polydopamine (GO/PDA) nano-interface-based microfluidic exosome analysis device was reported. It was shown that this nanostructured GO/PDA interface successfully suppresses non-specific exosome adsorption while significantly increasing exosome immuno-capture efficiency. An ultrasensitive exosome ELISA assay was developed based on this nano-interface and exhibits a very low detection limit [77].

In pancreatic ductal adenocarcinoma tissue (PDAC), the metalloprotease-disintegrin ADAM8 is abundantly expressed and inversely correlated with patient survival. EVs and cargo microRNAs (miRNAs) with the ADAM8 gene were found to be able to distinguish precursor lesions or PDAC from healthy controls. The presence of miR-720 and miR-451 was confirmed in 20 additional PDAC samples by EV cargo studies of miRNAs from the same blood samples. Upon comparison with healthy individuals, EVs from patients with PDAC or precursor lesions had a high enrichment of ADAM8 according to Fluorescence activated cell sorting analysis (*p* = 0.0005). More generally, this study showed that the presence of ADAM8 can activate an EV-based communication in the PDAC tumor microenvironment in a pro-oncogenic manner [78].

### 5.2. Alternating Current Electrokinetics (ACE)

Hinestrosa et al. [79] developed a machine learning method that can distinguish cancer patients from controls using multi-marker EV-protein measurements using an alternating current electrokinetics (ACE) platform to purify EVs from plasma. In this case-control pilot investigation, the sensitivity was 71.2% at 99.5% specificity when 184 control subjects were compared to 139 pathologically verified stage I and II cancer cases representing patients with pancreatic, ovarian, or bladder cancer. 95.5% of pancreatic, 74.4% of ovarian, and 43.8% of bladder cancer cases are diagnosed at stage I. Exosomes and other cellular nanoparticles can be separated from whole blood, plasma, or serum using a simple and quick technique devised by Lewis et al. [80]. These samples can then be examined for the presence of particular cancer-related protein and/or nucleic acid biomarkers. The entire test can be streamlined to be completed in less than 30 min with detection using directly-conjugated antibodies. Exosome isolation and biomarker detection are combined smoothly into a single, compact device by the ACE microarray devices integrated assay, paving the way for the creation of real sample-to-answer testing to track cancer biomarkers. The assay used in this study cannot differentiate between the two potential sources of high biomarker levels, entailing a raised biomarker-to-exosome ratio or an increase in the number of exosomes. The schematic representation and the outcomes in real time analysis is shown in Figure 8B.

Gaillard et al. [81] reported the contribution of biomolecules utilized as ligands in affinity-based biosensors for the detection and isolation of EVs. They mentioned that the short DNA pieces known as DNA aptamers, which mimic the actions of antibodies, are currently being produced and are thought to constitute the next generation of antibody-like ligands. These compounds have unmatched production benefits, extremely high air stability, affinity constants that are similar to antibodies, and compatibility with a wide range of organic solvents. Using EV-specific peptides to target EV membrane proteins and supplement other probes is another promising biological approach. Several types of biosensors, including electrochemical, optical, and microfluidics using both general probes, have utilized these various ligands.

### 5.3. Plasmon-Enhanced Fluorescence Detection

In order to convert EVs into clinically useful biomarkers, it is imperative to create novel EV molecular profiling technologies. Nanoplasmonic exosome (nPLEX), a technology created by Min et al. [82] is based on transmission surface plasmon resonance (SPR) across periodic nanohole gratings and can quickly and sensitively detect tumor-derived EVs from clinical samples [5]. They recently published the results of their next-generation nPLEX assay, which allows multiplexed single EV assessments of target membrane and intra-vesicular markers with increased sensitivity. For precise, multi-channel EV biomarker profiling, they specifically employed plasmon-enhanced fluorescence detection, which can magnify fluorescence signals utilizing plasmonic metallic nanostructures. Particularly, for low-abundance markers the plasmon enhancement makes single EV analysis more precise, sensitive and additional system improvement results in even greater signal augmentation [5].

### 5.4. Multiplexed and Other Platforms

**Figure 9:**
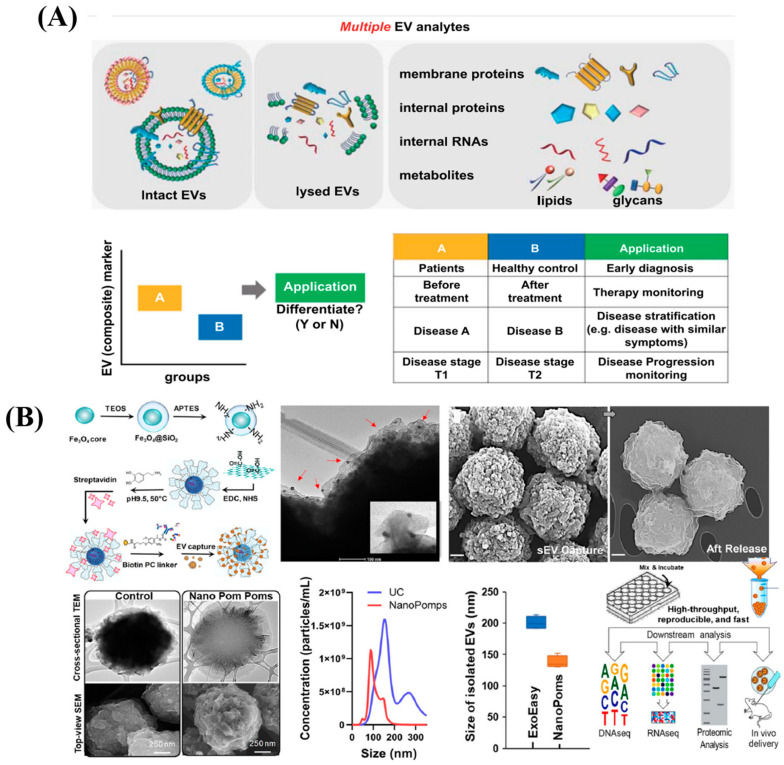
(**A**) Pictorial image of potential clinical applications of composite EV biomarkers. Re-used with permission from Ref. [83] and (**B**) represents the Nano pompoms fabrication for highly specific exosome isolation and multi-omic biomarker analysis. Re-used with permission from Ref. [84].

Jiang and team [83] evaluated the fundamental methods utilized to create multiplexing platforms for intact EVs and EV-derived proteins, RNAs, and metabolites as well as their application to clinical samples. As a result, there are four primary categories of EV multiplexed profiling strategies: chemical, physical, biological or nanoparticle-based coding. In actuality, one or more of these four methods or their combination are used to do this through bio-affinity-induced binding events between the receptor and the EV analyte. They claim that the key hurdles in the development of EV biomarkers are the low-throughput point of care devices for the detection of validated EV biomarkers and the high-throughput EV profiling platforms for screening several analytes from patients. It is also very important to look into multidimensional markers such as RNA and proteins in one device. This can minimize handling variations among various detection platforms significantly. In order to maximize the impact of multiplexed measures across large cohorts, advanced data processing is essential. To maximize the advantages of multi-purpose markers, such as multi-omics-based biomarkers, machine learning has been applied extensively. The use of sophisticated algorithms that can analyze the causal relationship between EV biomarkers and disease will make it easier to create composite marker patterns that function well. The pictorial image of potential clinical applications of composite EV biomarkers is shown in Figure 9A [83]. Peculiar flower pom-pom form and photo-click chemistry for specific marker defined capture and release of intact exosomes were found in innovative 3D-structured nanographene immunomagnetic particles (NanoPoms). According to a multi-omic exosome analysis of bladder cancer patient tissue fluids using the next-generation sequencing of somatic DNA mutations, miRNAs, and the global proteome, this particular exosome isolation approach results in the extended identification of targetable cancer biomarkers with better specificity and sensitivity. The produced exosomes by NanoPoms also display unique in vivo bio distribution patterns, emphasizing their very viable and essential quality. Figure 9B represents the Nano pom-poms fabrication for highly specific exosome isolation and multi-omic biomarker analysis [84].

### 5.5. EV in Immune System

EVs play a variety of roles in inflammatory processes and it can be secreted by all immune cell types that take part in inflammation [69,72]. It is also possible that EVs released by migratory inflammatory cells create persistent secondary chemotactic gradients (or “trails”) in the extracellular matrix for other cells to bind the components of the extracellular matrix. Numerous studies have shown that EVs produced by either non-inflammatory or inflammatory types of cell death have conflicting consequences when phagocytic cells take them up. Anti-tumor immunity is undoubtedly the most researched topic in the realm of EV-associated immunological responses. Through their actions on NK cells, T cells, DCs, macrophages, myeloid-derived suppressor cells (MDSCs), and regulatory B cells, tumor cell-derived EVs primarily decrease anti-tumor immune responses. Antitumor immunity also involves immune cell-derived EVs generated by DCs, T_reg_ cells, NK cells, B cells, and T cells that are associated with tumors.

The activation of a shift in macrophage polarization to an M2-type phenotype by EV-associated miR-145 through the downregulation of histone deacetylase-11 expression is a typical consequence of EVs produced from tumor cells. Furthermore, there is proof that tumor-derived EVs may unexpectedly affect cancer patients’ antiviral defense by transmitting tumor growth factor receptors to a specific subset of leukocytes. Tumor cell-derived EVs transport active EGFR molecules to host macrophages in epidermal growth factor receptor (EGFR)-positive lung cancers. EGFR then activates mitogen-activated protein kinase kinase 2 (MEKK2) to adversely control the antiviral immune response. The immunocompromised state of cancer patients may be partially explained by this process. The control of tumor-infiltrating T_reg_ cells and antitumor immunity is significantly influenced by EVs generated from tumor cells and CD300A of DCs. The control of tumor-infiltrating T_reg_ cells and antitumor immunity is significantly influenced by EVs generated from tumor cells and CD300A of DCs. Notably, adaptive “immunogenic stress” responses of tumor cells are triggered under unfavorable conditions in the tumor microenvironment, such as during hypoxia or nutritional restriction, which increases the release of EVs with an altered molecular composition. These EVs, which are produced from tumor cells, carry DAMPs such as HMGB1, HSPs, ATP, and mitochondrial DNA. By fostering an inflammatory environment, these DAMPs may facilitate immune identification of the tumor. Additionally, cancer-derived EVs carry tumor-associated antigens that, when taken up by APCs, may activate CD8+ T cells that are specific to the tumor. Stem cell-derived EVs with immunoregulatory effects are leading the way among the current EV-based immunotherapeutic strategies, mostly employing EVs produced from mesenchymal stem cells. These EVs not only promote tissue healing but also have a significant immunosuppressive potential. In the same way that tumor cell-derived EVs were discussed above, EVs derived from stem cells and progenitor cells also inhibit NK cell responses, DC maturation, and activation, induce M2-type macrophage polarization, support T_reg_ cell differentiation, and prevent B cell proliferation and differentiation [69].

### 5.6. Single Extracellular Vesicle

**Figure 10:**
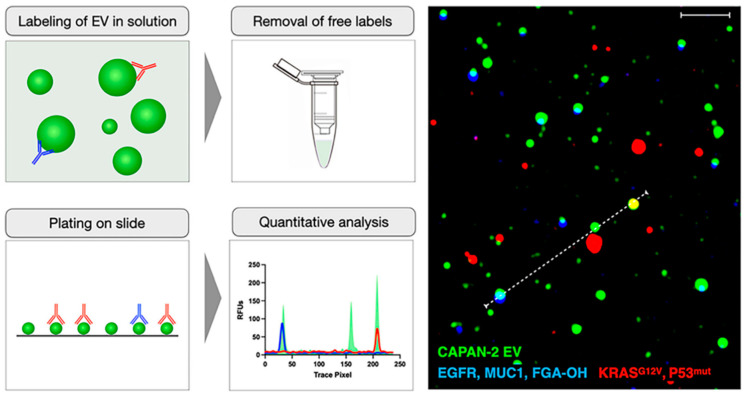
Synopsis of the sEVA technique. Re-used with permission from Ref. [86] (Copyright, 2022 Elsevier, reproduced with permission from Elsevier Ltd.).

Recently, for the first time, single EV analytical techniques with restricted multiplexed analysis were described [85]. A rising number of techniques are used nowadays, and the majority of them make use of the fundamental ideas behind fluorescence sensing, light scattering, or electron absorption. The significance of multi-parameter analysis, which combines EV detection with ctDNA mutation detection and other cancer biomarker measurement to improve diagnostic performance, has been further highlighted by recent research methodologies. All currently available clinically applicable single EV methods make use of fluorescently tagged antibodies. Three important techniques are a solution-based labelling single EV analysis (sEVA), a digital EV screening methodology (DEST), and a multiplexed analysis of single EV (MASEV). The most sensitive of them are probably sEVA and MASEV, which is probably needed for early cancer detection. Insight into vesicular heterogeneity within a sample and comparison of the EV protein make-up to that of the mother cell are two benefits of single EV analysis [86]. The protocol of the sEVA technique is shown in Figure 10.

## 6. Conclusions with Future Perspectives

In this review, we have detailed the different existing analytical techniques for cancer analysis, diagnostics and detection thorough molecular biomarkers. The cancer diagnosis is often based on the panel of genomic and proteomic biomarkers. The proteomic biomarkers have weak clinical performance and the original claims fail validation. Enzyme related proteins serve as important biomolecules for cancer biomarkers. However, only a limited number of protein biomarkers are available which will not completely network the clinical purpose. Furthermore, abundant protein found in human body which may interfere our analyte of interest, so automatically due to non-specific adsorption the sensitivity and selectivity is minimized. In the case of nucleic acid-based biomarkers, they are attractive due to their accuracy and possibility of simultaneous detection. However, the nuclease can breakdown under physiological conditions and become toxic and change the metabolism, which is a potential drawback.

On the other hand, EV-based multi-tumor screening test may vary depending upon the device’s intended application and the type of malignancy. The validation and practical translation of EV-based biomarkers now in the development will probably be delayed due to a lack of reliable EV isolation techniques suited for clinics. It is important to note that despite the realization of single-cell proteomics and sequencing, the translation of these technologies to single-EV measurements is attractive due to the traces of their contents. Sensitivity, probing ctEV to determine their organ of origin utilizing multiplexing techniques, and identification/separation of ctEV from relatively inactive against highly aggressive malignancies are the required essential enhancements in the existing single EV technologies.

In summary, a society free of cancer will result from the advancement of all biomarkers. On the basis of distributed datasets, the federated learning model can also be employed for cancer in remote locations. In contrast to traditional methods, biosensors are proven to be more affordable, quick, sensitive, and specialized possibilities that are essential for the early-stage cancer diagnostics for improved disease monitoring and therapy. The main difficulty is to minimize the size of the biosensors without sacrificing accuracy and portability for point-of-care diagnostics. The development of next-generation diagnostic methods by utilizing their binding affinity with other components is necessary to bring this decentralization testing on par with cancer detection and controlling non-specific adsorption issues. Moreover, lab-on-a-chip technology can be used to build advanced diagnostics, considerably reducing the challenge of exposure and transmission concerns in a non-invasive compact approach. Furthermore, multiplex biosensor arrays with the ability to simultaneously detect numerous biomarkers on a single chip should be realized. Such biosensors should also be compatible with microfluidics, biomarker pattern software and artificial intelligence programs. Using machine and deep learning approaches for extracting and categorizing the disease features, AI techniques play a vital role in early cancer prognosis and detection. Furthermore, there is a demand to detect head and neck related cancers because no work has been reported so far in that region.

## Figures and Tables

**Table 1:** List of protein biomarkers along with its related cancer type.

S. No	Name	Cancer Type
1.	AFP	Testicular cancer
2.	ß-hGC	Testicular cancer
3.	CA 19-9	Pancreatic cancer
4.	CA 125	Ovarian cancer
5.	CA 15.3	Breast cancer
6.	CA 27.9	Breast cancer
7.	CEA	Colorectal cancer
8.	FDP	Bladder cancer
9.	HE4	Ovarian cancer
10.	PSA	Prostate cancer
11.	TG	Thyroid cancer
12.	EGFR	Colorectal cancer
13.	KIT	Gastrointestinal cancer
14.	ER	Breast cancer
15.	PR	Breast cancer
16.	HER2-neu	Breast cancer
17.	NMP/22	Bladder cancer
18.	BTA	Bladder cancer
19.	Mw CEA	Bladder cancer

## Manuscript Number

CECRI/PESVC/Pubs/2023-015.
